# Micromechanical properties of canine femoral articular cartilage following multiple freeze-thaw cycles

**DOI:** 10.1016/j.jmbbm.2017.03.006

**Published:** 2017-07

**Authors:** Abby E. Peters, Eithne J. Comerford, Sophie Macaulay, Karl T. Bates, Riaz Akhtar

**Affiliations:** a Department of Musculoskeletal Biology, Institute of Ageing and Chronic Disease, University of Liverpool, The William Henry Duncan Building, 6 West Derby Street, Liverpool L7 8TX, UK; b Department of Mechanical, Materials and Aerospace Engineering, School of Engineering, University of Liverpool, The Quadrangle, Brownlow Hill, Liverpool L69 3GH, UK; c Institute of Veterinary Science, Leahurst Campus, University of Liverpool, Chester High Road, Neston, Wirral CH64 7TE, UK

**Keywords:** AFM, Atomic Force Microscopy, CSM, Continued Stiffness Measurement, E, Elastic Modulus, ECM, Extra Cellular Matrix, *G’,*, Shear Modulus, *G”*, Storage Modulus, PBS, Phosphate Buffered Solution, SD, Standard Deviation, Standard Error Mean (SEM), Canine, Femoral, Cartilage, Freezing, Nanoindentation

## Abstract

Tissue material properties are crucial to understanding their mechanical function, both in healthy and diseased states. However, in certain circumstances logistical limitations can prevent testing on fresh samples necessitating one or more freeze-thaw cycles. To date, the nature and extent to which the material properties of articular cartilage are altered by repetitive freezing have not been explored. Therefore, the aim of this study is to quantify how articular cartilage mechanical properties, measured by nanoindentation, are affected by multiple freeze-thaw cycles. Canine cartilage plugs (n = 11) from medial and lateral femoral condyles were submerged in phosphate buffered saline, stored at 3–5 °C and tested using nanoindentation within 12 h. Samples were then frozen at −20 °C and later thawed at 3–5 °C for 3 h before material properties were re-tested and samples re-frozen under the same conditions. This process was repeated for all 11 samples over three freeze-thaw cycles. Overall mean and standard deviation of shear storage modulus decreased from 1.76 ± 0.78 to 1.21 ± 0.77 MPa (*p* = 0.91), shear loss modulus from 0.42 ± 0.19 to 0.39 ± 0.17 MPa (*p*=0.70) and elastic modulus from 5.13 ± 2.28 to 3.52 ± 2.24 MPa (*p* = 0.20) between fresh and three freeze-thaw cycles respectively. The loss factor increased from 0.31 ± 0.38 to 0.71 ± 1.40 (*p* = 0.18) between fresh and three freeze-thaw cycles. Inter-sample variability spanned as much as 10.47 MPa across freezing cycles and this high-level of biological variability across samples likely explains why overall mean “whole-joint” trends do not reach statistical significance across the storage conditions tested. As a result multiple freeze-thaw cycles cannot be explicitly or statistically linked to mechanical changes within the cartilage. However, the changes in material properties observed herein may be sufficient in magnitude to impact on a variety of clinical and scientific studies of cartilage, and should be considered when planning experimental protocols.

## 1 Introduction

Articular cartilage is a viscoelastic heterogeneous material divided into layered zones with varying material properties and functionalities (Silver et al., 2002). The extracellular matrix (ECM) is heterogeneous in nature, where variations exist in composition, structure and vascularity at a micro-level. It is composed of proteoglycans, collagens and glycoproteins, which are all macromolecular components (Silver et al., 2002). Cartilage also contains chondrocytes that become embedded within the matrix, maturing and dividing to deposit new cartilage. Its primary function is to maintain a smooth surface allowing lubricated frictionless movement and to help transmit articular forces, therefore minimising stress concentrations across the joint (Nigg and Herzog, 2006).

Knowledge of material properties of cartilage is crucial to understanding its mechanical function and morpho-functional alterations that occur during ageing, disease and injury (Wen et al., 2012, Kleemann et al., 2005). Whilst valuable data in isolation, material property information is also crucial to other mechanical analyses, including computational models that attempt to predict *in vivo* joint behaviour (e.g. Wang et al., 2014, Guo et al., 2009, Pena et al., 2006). Material properties of articular cartilage ECM have been widely reported utilising varying testing, storage and preservation techniques (e.g. Shepherd and Seedhom, 1997, Kleemann et al., 2005, Wen et al., 2012). Specific testing techniques have changed over time and varied according to investigator preference and overall experimental goals. In general, however, all studies seeking to quantify the mechanical behaviour of biological tissues strive to maintain biological fidelity of the testing conditions in the experiment; for example testing fresh tissue samples under hydrated conditions that are representative of the internal environment of the studied organism (Brandt et al., 2010). However, accomplishing this may be challenging for numerous reasons including the need for transportation between dissection and testing locations, availability or failure of testing equipment and the desire to test large sample numbers from individual specimens thereby minimising tissue waste. In such circumstances it is standard practice to store and preserve samples, often requiring tissue to undergo one or more freeze-thaw cycles before mechanical tests can be carried out (e.g. Wilusz et al., 2013, Lau et al., 2008; Li et al., 2006).

Therefore in situations where logistical limitations prevent testing of fresh samples, it is beneficial to explore if preservation of tissues samples through freezing can be utilised without compromising mechanical properties. In recent years there have been a number of systematic investigations into the effects of multiple freeze-thaw cycles on the mechanical properties of ligaments and tendon (Huang et al., 2011, Moon et al., 2006, Woo et al., 1986). Although some variation between individual studies exists, these analyses suggest that ligament and tendon tissue can undergo a minimum of two freeze-thaw cycles before significant changes to their material properties occur, thereby providing important constraints on experimental designs involving these tissues. However, despite its fundamental importance to joint biomechanics, to the best of our knowledge, no such data exists exploring the effect of more than one freeze-thaw cycle on material properties of articular cartilage. The aim of this paper is therefore to quantify how articular cartilage mechanical properties are affected by multiple freeze-thaw cycles directly addressing this important gap in knowledge. Dynamic nanoindentation is used to determine the shear storage modulus (*G’*), shear loss modulus (*G”*), elastic modulus (*E*) and the loss factor (*tan δ*) of canine femoral condyle articular cartilage across three freeze-thaw cycles.

## 2 Materials and methods

### 2.1 Specimen preparation

**Fig. 1:**
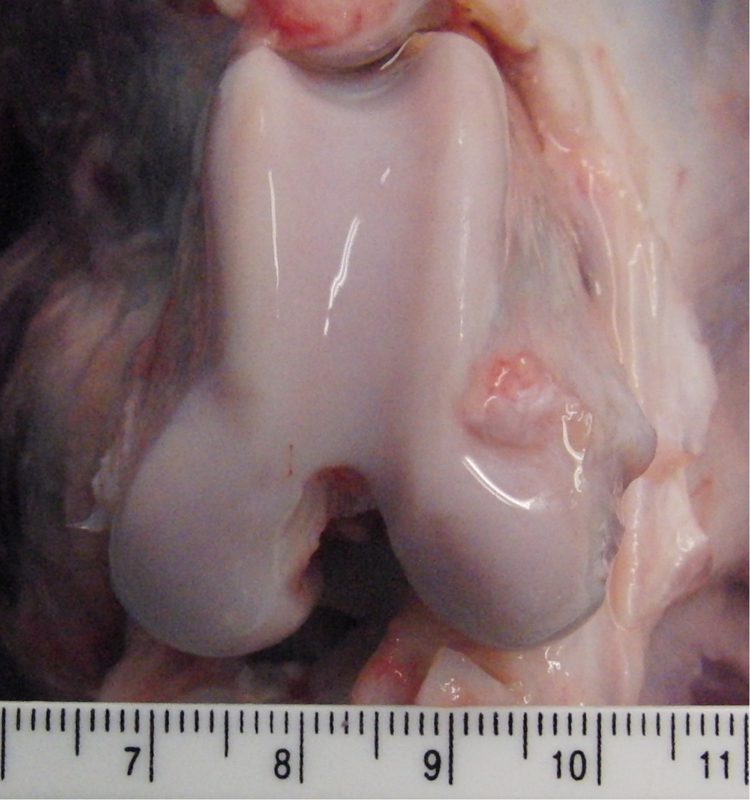
Photograph of the medial and lateral femoral condyle of the canine specimen to scale (cm), from which samples were harvested.

One disease free canine cadaveric knee joint from a skeletally mature Staffordshire Bull cross mix was dissected 36 h after being euthanized. Ethical permission for use of this cadaveric material was granted by the Veterinary Research Ethics Committee, University of Liverpool (VREC327). Healthy articular cartilage samples (n = 11) measuring < 1 cm^2^, were harvested from the medial and lateral bilateral femoral condyles (Fig. 1) using a low speed band saw (deSoutter Medical, Bucks, UK). Gross examination of the samples showed no sign of fibrillation or wear.

Following dissection, each of the 11 samples were submerged in phosphate buffered saline (PBS) and stored in cooled temperatures (3–5 °C) for up to 12 h until they were tested when still fresh using nanoindentation techniques, as detailed below. Following testing, all 11 samples were then frozen at −20 °C for up to 48 hours. Samples were then individually thawed for three hours at 3–5 °C and re-tested using the same nanoindentation protocol after having undergone one freeze-thaw cycle. This was completed within one hour and hydration of cartilage was maintained through constant exposure to PBS prior to and during testing (Brandt et al., 2010). This freeze-thaw procedure was repeated for three cycles and material properties of all 11 samples were measured after each freeze-thaw cycle. Samples were specifically thawed in cooled conditions (3–5 °C), as room temperatures have been shown to thaw cartilage samples too quickly and cause damage to the ECM (Szarko et al., 2010).

### 2.2 Nanoindentation testing

Cartilage samples underwent dynamic nanoindentation (G200 Nanoindenter, Keysight Technologies, Chandler, AZ, USA) equipped with an ultra-low load DCM-II actuator utilising a Continuous Stiffness Measurement (CSM) module to determine the micromechanical complex shear modulus.

**Fig. 2:**
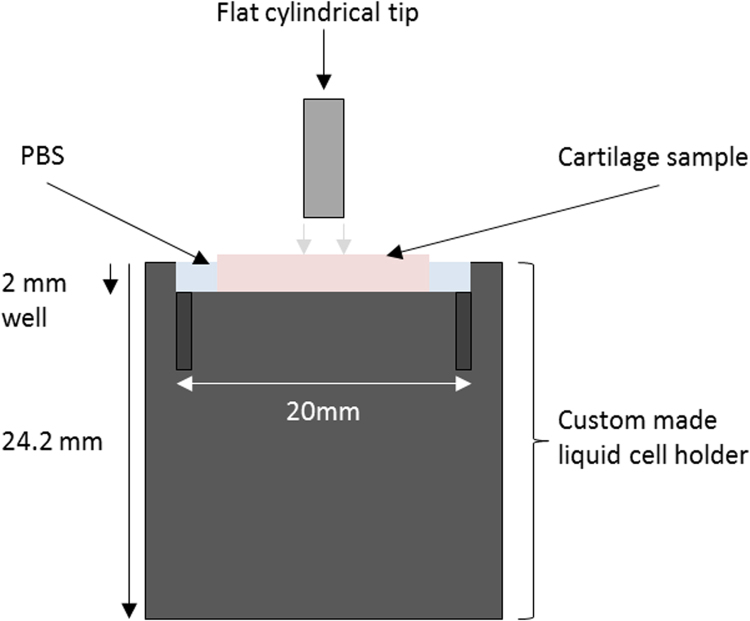
A schematic of the custom made liquid cell holder holding the cartilage sample and phosphate buffered saline (PBS).

Samples were mounted into a custom made liquid cell holder, with a 1 cm radius and 2 mm deep well, which could allow partial submersion of the samples in PBS during testing (Fig. 2). Samples were then examined under the built-in optical microscope to randomly select ten indent locations per sample (> 100 µm spacing between each indentation to avoid immediate overlap) totalling 110 measurements per cycle of freezing. Given that it was not possible to differentiate between microstructural features in the cartilage with the optical microscope, indentation sites were based on topographical homogeneity for accurate surface detection. Repetition or overlapping indentations in subsequent cycles of freezing was possible although it has previously been reported that there is no visible deformation of cartilage following low loads such as those experienced during nanoindentation when a recovery time is incorporated (Franke et al., 2011). Similarly to previous research investigating viscoelastic materials (e.g. Cheng et al., 2000, Jurvelin et al., 2000), a flat-ended cylindrical 100 µm punch tip (Synton-MDP Ltd, Nidau, Switzerland) was utilised as opposed to a sharp Berkovich tip which has been used in other studies testing cartilage (Hargrave-Thomas et al., 2015, Campbell et al., 2012, Franke et al., 2007, Gupta et al., 2005).

A Poisson's ratio of 0.46 (Jin and Lewis, 2004) was assumed for cartilage allowing the calculation of *G′*, *G′′* and the loss factor (i.e. ratio of *G′′* / *G′*) after each indentation. The theoretical basis is outlined in brief below and has been described in more detail previously (Herbert et al., 2008 and Herbert et al., 2009). Complex shear modulus (G*) is calculated by adding the shear storage modulus *G’* (real intrinsic elastic component) to the shear loss modulus *G”* (imaginary viscous component):

(1) $$G^{*}=\;G^{\prime }+\;{\mathrm{iG}}^{"}$$

Sneddon's analysis (Sneddon, 1965) is used to calculate the shear storage modulus using the Poisson's ratio (*v*), contact stiffness (*S*) and tip diameter (*D*), based on using a flat cylindrical punch:

(2) $$G^{\prime }=\frac{S\left(1-v\right)}{\left(2D\right)}$$

The above components along with contact damping (*Cw*) can be used to calculate the shear loss modulus: modulus:

(3) $$G^{"}=\frac{\mathrm{Cw}(1-v)}{\left(2D\right)}$$

Contact stiffness (*S*) is calculated by subtracting the instrument stiffness (*Ki*) from the total measured stiffness (*Ks*):

(4) S=Ks−Ki

Contact damping (*Cw*) is calculated by subtracting the instrument damping (*Ciw*) from the total measured damping (*Csw*):

(5) Cw=Csw−Ciw

The elastic modulus (*E*) was then calculated using the shear storage modulus (*G’*) and Poisson's Ratio (*v*) (Landau and Lifshitz, 1986):

(6) $$E=2G^{\prime \;}\left(1+v\right)$$

After the indenter head detected the surface of the sample, a pre-compression of 8 μm was applied until the indenter was fully in contact with the sample. The surface detection was determined by a phase shift of the displacement measurement. In order to accurately detect the surface, the phase shift was monitored over a number of data points which has previously been shown to be effective (Akhtar et al., 2016). Once the surface detection requirement was fulfilled over the predefined number of data points, the initial contact was determined from the first data point in the sequence. Once the indenter was fully in contact with the sample surface it vibrated at a fixed frequency of 110 Hz (the resonant frequency of the indenter) with 500 nm oscillation amplitude. Contact stiffness and damping were obtained through electromagnetic oscillation sequences. The initial oscillation measured instrument stiffness and damping and these were subtracted from the total measurement to obtain the contact response. Material properties were then obtained during the second oscillation.

After each indentation, the tip was cleaned to prevent any transfer of biological material to the subsequent indentation site which may affect measurements. This was achieved by indenting an adjacent sample holder which was mounted with 3 M double-sided Scotch tape. This method was found to be effective at cleaning the tip without picking up any residue from the Scotch tape. Following testing of each sample, further indents were made on fused silica with the test sites remaining free of any residue, hence confirming that the tip was clean before further cartilage testing.

### 2.3 Statistical analysis

2.3

An a-priori power analysis was performed using G*Power software (Faul et al., 2007) which specified a total of eight samples would be required to distinguish an effect size of 0.8 with α error probability of 0.05 and power of 0.95 across four groups of testing parameters. Statistical analysis of *G’*, *G”* and *E*, as well as the loss factor, were conducted using a repeated measures ANOVA in SPSS (SPSS software, Version 22.0, SPSS, Inc., Chicago, IL), specifically Mauchly's Test of Sphericity, after which a Bonferroni post-hoc test was performed if results were significant, producing pairwise comparisons. Individual sample means were analysed after each cycle of freezing, as well as the means of all samples combined, to give a whole specimen analysis.

## 3 Results

### 3.1 Overall Trends

**Fig. 3:**
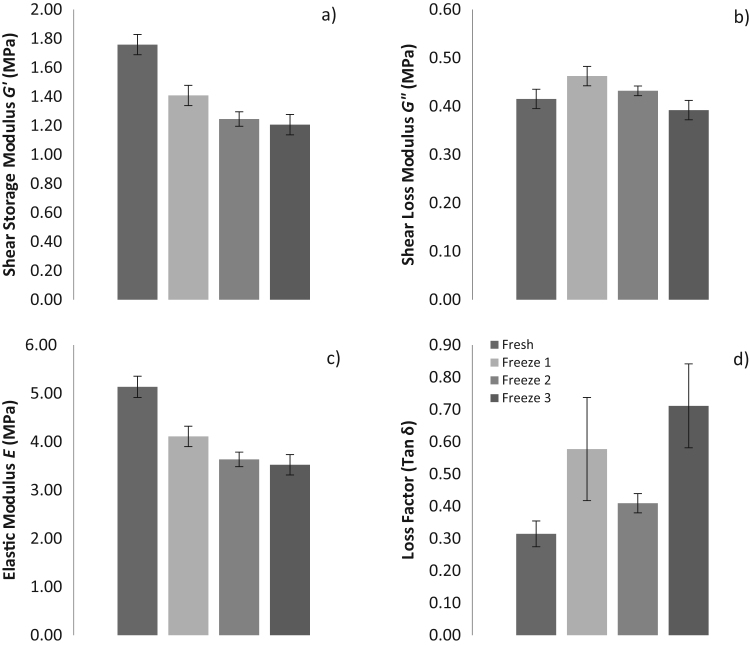
a) Mean shear storage modulus (*G’*), b) Shear loss modulus (*G”*), c) Elastic modulus (*E*) and the d) Loss factor for all samples combined during different storage and freezing conditions. Error bars represent the standard error of mean (SEM).

The overall mean *G’*, *G”*, *E* and loss factor for all 11 samples combined for the different cycles are presented in Fig. 3. Shear modulus (*G’*) decreased from 1.76 ± 0.78, 1.41 ± 0.77, 1.25 ± 0.54 to 1.21 ± 0.77 MPa (mean ± standard deviation (SD)) between fresh samples and samples tested after one, two and three freeze-thaw cycles respectively (Fig. 3a). Shear loss modulus (*G”*) increased from 0.42 ± 0.19 to 0.46 ± 0.18 MPa (mean ± SD) between fresh and one freeze-thaw cycle, but then decreased to 0.43 ± 0.15 and 0.39 ± 0.17 MPa following two and three freeze-thaw cycles respectively (Fig. 3b). Elastic Modulus (*E)* were 5.13 ± 2.28, 4.11 ± 2.25, 3.64 ± 1.57 and 3.52 ± 2.24 MPa (mean ± SD) during fresh, one, two and three freeze-thaw cycles respectively (Fig. 3c). The mean and SD of the loss factor changed throughout each cycle from 0.31 ± 0.38, 0.58 ± 1.66, 0.41 ± 0.26 and 0.71 ± 1.40 when using a mean of all 11 samples during fresh, one, two and three freeze-thaw cycles respectively (Fig. 3d). Changes in the values for *G’*, *G”*, *E* and the loss factor, across freeze-thaw cycles were not found to be statistically significant (Mauchley's Test of Sphericity, *p* = 0.91, *p* = 0.70, *p* = 0.20, *p* = 0.18 respectively).

### 3.2 Inter-Sample Variability

Numerical results for individual samples are tabulated in Table 1, Table 2, Table 3, Table 4. Repeated freeze-thaw cycles led to some significant differences in *G’* (*p* = 0.016) and *E* (*p* = 0.019) across individual samples but no differences in *G”* (*p* = 0.122) or the loss factor (*p* = 0.178). Bonferroni post-hoc pairwise comparisons showed between freeze-thaw cycle effects on the individual sample mean *G’* and *E* were not statistically significant between fresh and one freeze-thaw cycle (*p* = 0.45), one freeze-thaw and two freeze-thaw cycles (*p* = 1.00), and two freeze-thaw and three freeze-thaw cycles (*p* = 1.00). Further post-hoc pairwise comparison was not necessary for *G”* or the loss factor, as these were not statistically significant.

A high degree of variability in each mechanical property was observed both within and between the 11 discrete samples analysed at each freeze-thaw cycle, as indicated by high standard deviations about the overall mean values (as listed above) and the substantial absolute ranges of individual sample means and coefficient of variation (CoV) (Table 1, Table 2, Table 3, Table 4). For example, the *E* value in an individual sample in the same cycle of fresh testing varied by as much as 10.47 MPa equivalent to a change of up to 96.29% of the overall mean value on one occasion (Table 3). Across the 11 samples tested, *E* varied by as much as 14.73 MPa or equivalent to a 188.89% change to the overall mean within the same cycle of freezing (mean / SD) seen in Table 3. Inter-sample variation was such that in some instances individual samples exhibited changes in mechanical properties across freeze-thaw cycles that differed qualitatively from the overall mean trends (Fig. S1).

## 4 Discussion

This study provides the first systematic investigation of the effects of multiple freeze-thaw cycles on the mechanical properties of articular cartilage. Szarko et al., (2010) compared the mechanical properties of canine femoral articular cartilage stored at −20 °C, −80 °C and snap frozen in liquid nitrogen using indentation techniques. They found that with rapid thawing (37.5 °C) and exposure to PBS, both −20 °C and −80 °C can be used as reliable preservation methods for one freeze-thaw cycle as this produced results consistent with those from fresh samples. However, snap freezing tissue can cause ice crystallisation to form on the sample and therefore compromises the integrity of the tissue. Further research (Moore and Burris, 2015) also considered the effects of one freeze-thaw cycle at −80 °C on the mechanical properties of bovine femoral and tibial articular cartilage in comparison to fresh samples. Using a custom made indenter samples were exposed to PBS to maintain hydration and thawed at room temperature. No significant change in material properties was found with a tensile modulus of 4.1 ± 2.2 MPa for fresh samples and 4.5 ± 2.4 MPa for frozen samples (Moore and Burris, 2015). However, individual samples were randomly assigned to a fresh or frozen cohort and testing was not repeated on the same sample. Therefore results did not account for biological variability that may exist spatially within one specimen or cadaver. Wilusz et al. (2013) used two freeze thaw cycles at −20 °C of human femoral articular cartilage prior to atomic force microscopy (AFM)-based indentation. Justification for using two freeze-thaw cycles was recommended by Athanasiou et al. (1991) who established this aspect of the protocol on anecdotal unpublished data. Samples were exposed to PBS to maintain hydration and results from healthy cartilage ECM presented an *E* of 491 kPa. However in this study, a comparison to fresh samples was not made therefore what effect two freeze-cycles had on the material properties is unknown (Wilusz et al., 2013).

Our research study demonstrated that mean cartilage *G’* and *E* for the joint overall showed a sharp decreasing trend after one cycle of freezing, although this reduction appeared to lessen following two and three freeze-thaw cycles, despite not reaching statistical significance (Fig. 3). Interestingly *G”* and the loss factor showed no such trends and both increased and decreased during various cycles of freezing (Fig. 3). The loss factor in particular showed high standard error mean (SEM) (Fig. 3) in comparison to other parameters. When analysing the SD it appears that there is no consistent trend or change in *G’* and *E* where values both increase and decrease in various cycles of freezing (Table 1, Table 3). With the exception of two outliers *G”* and the loss factor SD remains unchanged during all cycles of freezing (Table 2, Table 4).

Systematic testing of articular cartilage across multiple freeze-thaw cycles in our study shows that samples can undergo three freezing cycles without statistically significant changes to material properties when handled and stored correctly (Fig. 3). These results therefore provide some support for the use of freezing as a method of preservation of cartilage where material properties are required to remain unchanged for mechanical testing. However the authors note that a number of changes in individual mean material properties for the joint were observed here (Fig. S1), and although these fell below thresholds of statistical significance in this study they may represent meaningful magnitudes in the context of other studies. For example, the overall mean *E* showed relatively large decreases with increasing number of freeze thaw cycles such that the values decreased by 1.02 MPa (one freeze-thaw), 0.47 MPa (two freeze-thaw) and 0.12 MPa (three freeze-thaw) of the mean value compared to fresh samples. Such relative changes in magnitude may well be extremely important in the context of comparative studies such as comparison of material properties between cohorts of different age and/or disease status (Wen et al., 2012, Kleemann et al., 2005, Franz et al., 2001) and computational modelling studies of joint biomechanics (Mononen et al., 2012, Pena et al., 2007, Blankevoort et al., 1991). Kleemann et al., (2005) researched the differences in cartilage material properties obtained from human tibial plateau samples and found that changes of as little as 0.1 MPa or 20% can be found between grade one and grade two osteoarthritic samples (graded by the International Cartilage Repair Society). Furthermore, in a human knee finite element model sensitivity analysis by Li et al. (2001) the material properties of cartilage were varied between 3.5 and 10 MPa, to understand the effect on joint contact stresses. Results showed that magnitude changes had substantial effects on the functional predictions of the model, specifically that *E* linearly increased with peak contact stresses and a Poisson's ratio increase significantly increased peak von Mises stress and hydrostatic pressure in the knee joint cartilage.

Given the absolute and relative changes in overall material properties measured across freeze-thaw cycles (Fig. 3), it may be preferable for experiments seeking to test multiple tissue types from the same cadaver to prioritise cartilage for fresh testing (or minimal freeze-thaw cycles), particularly given that previous research has suggested that other joint tissues are relatively insensitive to freezing (Jung et al., 2011, Huang et al., 2011, Moon et al., 2006, Woo et al., 1986). For example, Jung et al., (2011) concluded that the human patella-tendon can be exposed to eight freeze-thaw cycles, without compromising mechanical properties; provided testing conditions and tissue handling are approached with great care. This protocol involved allowing samples to re-freeze for a minimum of 6 h and thaw at room temperature for 6 h with exposure to saline. Furthermore, a study has shown the human flexor digitorum superficialis and flexor pollicis longus can undergo three freeze-thaw cycles before the integrity of their material properties is compromised. In addition freeze-thawing over five times also results in decreased mechanical and structural behaviour (Huang et al., 2011). Other studies focusing on ligaments include Woo et al. (1986) who explored the mechanical properties of the rabbit medial collateral ligament (MCL) following one prolonged freezing cycle and concluded that this has no effect when compared to fresh samples. Moon et al. (2006) also used the rabbit MCL to determine the effect when two freeze-thaw cycles and likewise concluded that no apparent changes to material properties occurred when compared to fresh samples. Therefore most published studies are in agreement that at least two freeze-cycles, under the correct handling and storage conditions, allow ligament and tendon samples to remain mechanically unchanged (Jung et al., 2011, Huang et al., 2011, Moon et al., 2006, Woo et al., 1986).

The modulus values obtained within this study fall within the range of those reported in the literature for other mammalian femoral condylar articular cartilage. Shepherd and Seedhom (1999) and Wilusz et al. (2013) reported a range of *E* from 0.1 to 18.6 MPa for human femoral condyle articular cartilage, although Moore and Burris (2015) reported lower values of 0.62 ± 0.10 MPa for bovine stifle cartilage. In our study mean values for *E* lie between 0.56 and 7.62 MPa, falling within this range already reported; however in both the literature and the current study there is a high variability of modulus. More specifically, previous canine research has found an *E* of 0.12 ± 0.10 MPa (Leroux et al., 2001), and 0.385–0.964 MPa (Jurvelin et al., 2000) when samples have undergone indentation testing following one freeze cycle. These values are generally lower than those reported in our study and have smaller absolute variability. Previous canine cartilage studies have reported CoV's of up to 23.61% (Jurvelin et al., 2000), which although being quite considerable are much lower than the CofV's reported here up to 96.3% for *G’* and 114.29% for *G”* (Table 1, Table 2, Table 3, Table 4). Although the current data is more variable than previous canine research, it should be noted that it is less variable than the human studies discussed above.

Cartilage is a highly heterogeneous material and therefore some variability of modulus is widely expected and accepted (e.g. Jurvelin et al., 2000); however differences seen in the current study as compared to other studies in the literature may be as a result of the frequency-dependent properties of cartilage. Higher frequencies have been shown to increase *G’* (Pearson and Espino, 2013) and *E* (Taffetani et al., 2015); however *G”* remains unaffected (Pearson and Espino, 2013). In our study, 110 Hz was selected for the testing because it is the resonant frequency of the indenter and thus most sensitive frequency for the surface detection. In other studies in the literature, a range of frequencies have been used including 0.5 Hz (Taffetani et al., 2015), 10 Hz (Franke et al., 2011) and much higher frequencies up to 200 Hz (Taffetani et al., 2015) and 250 Hz (Franke et al., 2011) where dynamic nanoindentation (Franke et al., 2011) and mechanical analysis methods were also utilised (Taffetani et al., 2015). Although high frequencies may account for increases in *G’* when compared to other canine studies (Leroux et al., 2001; Jurvelin eta l., 2000), the most important comparison is that seen between each freeze cycle, where frequency used remained standardised throughout testing cycles.

Additional limitations to the current study which may also affect variability include indenting sites affected by preceding measurements; however it has been suggested that low load indentation has been shown to cause no visible deformation of samples (Franke et al., 2011). Although some variability may be expected from the nanoindentation technique used in the current study, we have found that it yields highly repeatable data on other compliant materials which have a more homogenous structure than cartilage e.g. on a type of ballistic gelatine (Perma-Gel) the CoV for the elastic modulus was 3.3% following ten indentation tests (Moronkeji et al., 2016).

As the nanoindenter was unable to differentiate between cellular and non-cellular substance, the current study is subject to high variability in results depending on the exact material tested, limiting interpretation of changes to modulus. Other studies have attempted to differentiate the material properties of cartilage sub-components using AFM and found variation between *E* of the peri- (0.1 MPa) and extra cellular matrix (0.3 MPa) (Wilusz et al., 2013). However soft tissues are often dehydrated during AFM testing and maintaining hydration can be challenging (Wen et al., 2012).

With these considerations in mind, future research could aim to accurately assess the effect of freezing on articular cartilage by first repeatedly indenting the same site of a fresh sample to fully understand the effect and variability of material properties seen in an identical position. Then secondly, indenting an identical position following multiple freeze-thaw cycles, aided by marking an area of the cartilage and noting at which exact position the sample was tested to understand the effect of freezing.

## 5 Conclusion

In summary, the results of this study suggest that three freeze-thaw cycles do not have a statistically significant effect on the overall ‘whole-joint’ material properties of canine femoral condyle cartilage samples provided the correct handling, storage and hydration of the tissue are maintained throughout preparation and testing. However, relative changes in mean material properties are observed and the failure to reach thresholds for statistical significance is likely the product of high biological variability across the joint. Therefore the changes in material properties observed over multiple freeze-thaw cycles may be sufficient to significantly impact on certain comparative or functional studies, such as finite element modelling, where subtle changes in material properties can indeed modify the true behaviour of articular cartilage under mechanical stress. Changes in material properties reported here should be considered when planning experimental protocols, as they may be sufficient in magnitude to impact on clinical or scientific cartilage studies.

## Conflict of interest

There are no conflicts of interest to declare.

## Figures and Tables

**Table 1:** Mean ± standard deviation (SD), standard error mean (SEM) and coefficient of variation (CoV) for Shear storage modulus (MPa) for each tested sample during each cycle of freezing.

Shear Storage Modulus *G’* (MPa)												
	Fresh			Freeze 1			Freeze 2			Freeze 3		
Sample	Mean±SD	SEM	CoV%	Mean±SD	SEM	CoV%	Mean±SD	SEM	CoV%	Mean±SD	SEM	CoV%
1	2.57±0.39	0.12	15.17	2.61±0.28	0.09	10.73	1.24±0.42	0.13	33.87	1.65±0.45	0.14	27.27
2	1.11±0.13	0.04	11.71	1.16±0.12	0.04	10.34	1.04±0.43	0.14	41.35	1.29±0.13	0.04	10.08
3	2.58±1.05	0.33	40.70	0.77±0.58	0.18	75.32	0.76±0.50	0.16	65.79	0.54±0.52	0.16	96.30
4	2.22±0.26	0.08	11.71	2.20±0.35	0.11	15.91	1.64±0.24	0.08	14.63	2.32±0.65	0.21	28.02
5	1.05±0.47	0.15	44.76	1.04±0.53	0.17	50.96	1.06±0.22	0.07	20.75	0.19±0.15	0.05	78.95
6	1.72±0.37	0.12	21.51	0.70±0.21	0.07	30.00	1.36±0.22	0.07	16.18	1.38±0.19	0.06	13.77
7	2.07±0.21	0.07	10.14	2.12±0.12	0.04	5.66	1.25±0.12	0.04	9.60	1.84±0.10	0.03	5.43
8	2.41±0.28	0.09	11.62	1.85±0.24	0.08	12.97	1.85±0.22	0.07	11.89	1.40±0.79	0.25	56.43
9	1.31±0.17	0.05	12.98	1.12±0.12	0.04	10.71	0.79±0.15	0.05	18.99	0.22±0.02	0.01	9.09
10	1.70±0.55	0.17	32.35	1.63±0.58	0.18	35.58	2.10±0.45	0.14	21.43	1.64±0.50	0.16	30.49
11	0.60±0.39	0.12	65.00	0.29±0.17	0.05	58.62	0.61±0.07	0.02	11.48	0.79±0.12	0.04	15.19

**Table 2:** Mean ± standard deviation (SD), standard error mean (SEM) and coefficient of variation (CoV) for Shear loss modulus (MPa) for each tested sample during each cycle of freezing.

Shear Loss Modulus *G”*(MPa)												
	Fresh			Freeze 1			Freeze 2			Freeze 3		
Sample	Mean±SD	SEM	CoV%	Mean±SD	SEM	CoV%	Mean±SD	SEM	CoV%	Mean±SD	SEM	CoV%
1	0.54±0.06	0.02	11.11	0.62±0.08	0.03	12.90	0.44±0.13	0.04	29.55	0.53±0.08	0.02	15.09
2	0.24±0.02	0.01	8.33	0.31±0.02	0.01	6.45	0.25±0.09	0.03	36.00	0.28±0.02	0.01	7.14
3	0.42±0.48	0.15	114.29	0.48±0.18	0.06	37.50	0.49±0.12	0.04	24.49	0.49±0.17	0.05	34.69
4	0.60±0.07	0.02	11.67	0.74±0.09	0.03	12.16	0.53±0.05	0.02	9.43	0.60±0.10	0.03	16.67
5	0.37±0.14	0.04	37.84	0.42±0.14	0.04	33.33	0.45±0.07	0.02	15.56	0.06±0.05	0.01	83.33
6	0.40±0.07	0.02	17.50	0.38±0.03	0.01	7.89	0.37±0.03	0.01	8.11	0.33±0.03	0.01	9.09
7	0.49±0.02	0.01	4.08	0.58±0.01	0.00	1.72	0.37±0.03	0.01	8.11	0.43±0.02	0.01	4.65
8	0.45±0.03	0.01	6.67	0.38±0.04	0.01	10.53	0.39±0.03	0.01	7.69	0.50±0.18	0.06	36.00
9	0.40±0.03	0.01	7.50	0.57±0.06	0.02	10.53	0.68±0.03	0.01	4.41	0.39±0.02	0.01	5.13
10	0.46±0.11	0.03	23.91	0.47±0.11	0.04	23.40	0.58±0.07	0.02	12.07	0.48±0.10	0.03	20.83
11	0.19±0.06	0.02	31.58	0.13±0.03	0.01	23.08	0.21±0.02	0.01	9.52	0.22±0.01	0.00	4.55

**Table 3:** Mean ± standard deviation (SD), standard error mean (SEM) and coefficient of variation (CoV) for Elastic modulus (MPa) for each tested sample during each cycle of freezing.

Elastic Modulus *E* (MPa)												
	Fresh			Freeze 1			Freeze 2			Freeze 3		
Sample	Mean±SD	SEM	CoV%	Mean±SD	SEM	CoV%	Mean±SD	SEM	CoV%	Mean±SD	SEM	CoV%
1	7.52±1.14	0.36	15.16	7.62±0.83	0.26	10.89	3.61±1.22	0.38	33.80	4.83±1.32	0.42	27.33
2	3.24±0.39	0.12	12.04	3.39±0.35	0.11	10.32	3.04±1.27	0.40	41.78	3.76±0.39	0.12	10.37
3	7.55±3.07	0.97	40.66	2.24±1.68	0.53	75.00	2.22±1.46	0.46	65.77	1.57±1.51	0.48	96.18
4	6.48±0.75	0.24	11.57	6.42±1.02	0.32	15.89	4.80±0.71	0.22	14.79	3.79±1.89	0.60	49.87
5	3.08±1.38	0.44	44.81	3.04±1.55	0.49	50.99	3.10±0.65	0.21	20.97	0.56±0.44	0.14	78.57
6	5.01±1.09	0.34	21.76	2.05±0.63	0.20	30.73	3.97±0.65	0.21	16.37	4.04±0.56	0.18	13.86
7	6.04±0.61	0.19	10.10	6.19±0.36	0.11	5.82	3.65±0.35	0.11	9.59	5.37±0.31	0.10	5.77
8	7.03±0.80	0.25	11.38	5.39±0.70	0.22	12.99	5.39±0.63	0.20	11.69	4.09±2.31	0.73	56.48
9	3.83±0.49	0.15	12.79	3.28±0.34	0.11	10.37	2.31±0.43	0.14	18.61	0.66±0.07	0.02	10.61
10	4.97±1.60	0.51	32.19	4.75±1.70	0.54	35.79	6.13±1.30	0.41	21.21	4.79±1.46	0.46	30.48
11	1.75±1.15	0.36	65.71	0.84±0.49	0.16	58.33	1.77±0.21	0.07	11.86	2.29±0.34	0.11	14.85

**Table 4:** Mean ± standard deviation (SD), standard error mean (SEM) and coefficient of variation (CoV) for Loss factor for each tested sample during each cycle of freezing.

Loss Factor												
	Fresh			Freeze 1			Freeze 2			Freeze 3		
Sample	Mean±SD	SEM	CoV%	Mean±SD	SEM	CoV%	Mean±SD	SEM	CoV%	Mean±SD	SEM	CoV%
1	0.21±0.03	0.01	14.29	0.24±0.01	0.00	4.17	0.36±0.04	0.01	11.11	0.34±0.11	0.04	32.35
2	0.22±0.01	0.00	4.55	0.27±0.01	0.00	3.70	0.25±0.03	0.01	12.00	0.22±0.01	0.00	4.55
3	0.21±0.14	0.04	66.67	2.46±5.33	1.69	216.67	0.85±0.46	0.14	54.12	2.02±2.41	0.76	119.31
4	0.27±0.01	0.00	3.70	0.34±0.03	0.01	8.82	0.33±0.03	0.01	9.09	0.27±0.04	0.01	14.81
5	0.36±0.05	0.02	13.89	0.45±0.13	0.04	28.89	0.43±0.06	0.02	13.95	0.31±0.01	0.00	3.23
6	0.24±0.02	0.01	8.33	0.61±0.24	0.07	39.34	0.28±0.04	0.01	14.29	0.24±0.02	0.00	8.33
7	0.24±0.02	0.01	8.33	0.27±0.01	0.00	3.70	0.30±0.01	0.00	3.33	0.24±0.00	0.00	0.00
8	0.19±0.01	0.00	5.26	0.21±0.01	0.00	4.76	0.21±0.01	0.00	4.76	1.83±3.42	1.08	186.89
9	0.31±0.04	0.01	12.90	0.51±0.07	0.02	13.73	0.88±0.12	0.04	13.64	1.76±0.11	0.04	6.25
10	0.28±0.05	0.02	17.86	0.31±0.06	0.02	19.35	0.29±0.04	0.01	13.79	0.30±0.04	0.01	13.33
11	0.93±1.12	0.35	120.43	0.68±0.62	0.20	91.18	0.34±0.02	0.01	5.88	0.29±0.04	0.01	13.79
