# Supplementary material for: Micromechanical properties of canine femoral articular cartilage following multiple freeze-thaw cycles
Source: J Mech Behav Biomed Mater. 2017 Jul;71:114–21. doi: 10.1016/j.jmbbm.2017.03.006 (PMC5429396; doi:10.1016/j.jmbbm.2017.03.006)
Supplement: Supplementary file 1 — Supplementary material [file mmc1.docx]

Supplementary Materials

1. Spines of the *Diodon hystrix* and *Diodon holocanthus*

##
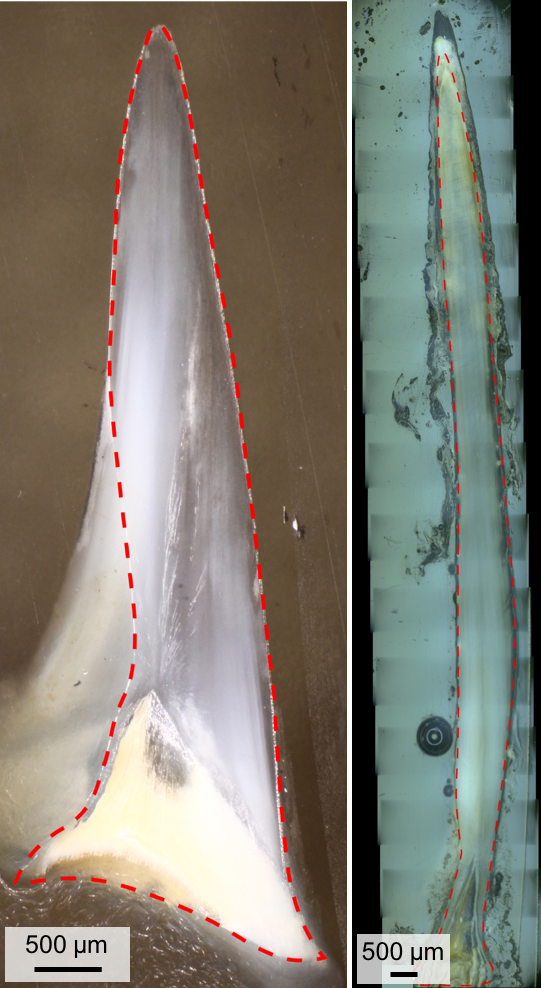


**Supplementary Figure 1**. (a) *Diodon holocanthus* and (b) *Diodon hystrix* spine cross-sections outlined in red. Both spines have a stiff spinous process and a soft region in the spine base.

1. Derivation of Equations for a Beam with a Uniform Cross-section and a Tapered Beam with Circular Cross-Sections

Consider a cantilever beam with uniform circular cross-section as shown in Figure 13a. The moment at any location when there is a point load at the end of the cantilever beam is given by

|  | $M\left( x \right)=-P\left( L-x \right)$ | (S1) |
| --- | --- | --- |

where *M* is the moment at position *x*, *L* is the length of the beam, and *P* is the point load at the end of the beam. The moment curvature relation is

|  | $M\left( x \right)=-EI\frac{d^{2}v\left( x \right)}{dx^{2}}.$ | (S2) |
| --- | --- | --- |

where *E* is Young’s modulus, *v* is vertical deflection (positive in the negative y-direction), and *I* is second moment of inertia. After integration and rearrangement,

|  | $E=\frac{64PL^{3}}{3{\pi v}_{max}d^{4}} .$ | (S3) |
| --- | --- | --- |

Since the porcupine fish spine is mostly mineral, it is expected to fail under tension rather than compression. The stress in a cantilever beam is

|  | $\sigma\left( x \right)=-\frac{M\left( x \right)y\left( x \right)}{I}$ | (S4) |
| --- | --- | --- |

The maximum tensile stress occurs at $\text{y}_{\text{max}}=d/2$. Substituting the expressions for the second moment of inertia and the maximum moment results in

|  | $\sigma_{max}=\frac{32 PL}{\pi d^{3}}$ | (S5) |
| --- | --- | --- |

From Hooke’s Law, $\text{ε}_{\text{max}}$ is the $\text{σ}_{\text{max}}$ divided by the *E, so that*

|  | $\varepsilon_{max}=\frac{\sigma_{max}}{E}=\frac{3v_{max}d}{2L^{2}}$ | (S6) |
| --- | --- | --- |

A tapered cantilever beam with constant diameter change and circular cross-section with smaller diameter *d_A_* at the end of the beam and the larger diameter *d_B_* at the base of the beam is shown in Figure 13b. The expression for strain energy ($U$) is:

|  | $U=\int_{0}^{L} \frac{M^{2}(x)}{2E I(x)}dx$ | (S7) |
| --- | --- | --- |

and *I*$\left( \text{x} \right)$is expressed as

|  | $I\left( x \right)=I_{B}\left( 1+\frac{\left( \beta-1 \right)}{L}x \right)^{4}$, $I_{B}=\frac{\pi{d_{B}}^{4}}{64}$ | (S8) |
| --- | --- | --- |

where *β* = *d_A_*/*d_B_* < 1, and $\text{I}_{\text{B}}$ is the second moment of area of the end of the beam with diameter $d_{B}$.

The maximum displacement of the tip of the tapered beam $\text{v}_{\text{max}}$ is given by Castigliano’s theorem as

|  | $v_{max}=\frac{\partial U}{\partial P}=\frac{\partial}{\partial P}\int_{0}^{L} \frac{M^{2}(x)}{2E I(x)}dx=\int_{0}^{L} \frac{M\left( x \right)\frac{\partial M}{\partial P}}{E I(x)}dx.$ | (S9) |
| --- | --- | --- |

The derivative of *M*$\left( \text{x} \right)$ with respect to $\text{P}$ is $-\left( \text{L}-\text{x} \right).$ Substituting this and Eqns. (S1), and (S8) into Eqn. (S9)

$$v_{max}=\frac{P}{E I_{B}}\int_{0}^{L} \left( \frac{L^{2}}{\left( 1+\frac{(\beta-1)}{L}x \right)^{4}}-\frac{2L x}{\left( 1+\frac{(\beta-1)}{L}x \right)^{4}} \right.+\left. \frac{x^{2}}{\left( 1+\frac{\left( \beta-1 \right)}{L}x \right)^{4}} \right)dx$$

and after integration and rearrangement:

|  | $E =\frac{64 PL^{3}}{3\pi\beta{d_{B}}^{4}v_{max}}.$ | (S10) |
| --- | --- | --- |

The maximum stress for the tapered beam can be derived from Eqn. (S4). Assuming a linear change in diameter along the length of the spine, *y*(*x*) can be represented as a function of the diameter of the beam cross-section *d*(*x*),

|  | $y\left( x \right)=\frac{d(x)}{2}=\frac{d_{B}}{2}\left( 1+\frac{\left( \beta-1 \right)}{L}x \right).$ | (S11) |
| --- | --- | --- |

Substituting Eqns. (S1), (S8) and (S11) into Eqn. (S4) and reducing,

|  | $\sigma\left( x \right)=\frac{32P\left( L-x \right)}{\pi{d_{B}}^{3}\left( 1+\frac{\left( \beta-1 \right)}{L}x \right)^{3}}.$ | (S12) |
| --- | --- | --- |

To find $\text{σ}_{\text{max}}$, the roots of *σ*$\left( \text{x} \right)=0$ must be found by taking the derivative of *σ*$(\text{x})$ with respect to *x* and setting it equal to 0. Simplifying results, the following expression is found

|  | $x=\frac{L\left( 3\beta-2 \right)}{2\left( \beta-1 \right)}$ | (S13) |
| --- | --- | --- |

where 0 < *β* < 2/3. Substituting Eqn. (S13) into Eqn. (S12)

|  | $\sigma_{max}=\frac{128PL}{27\pi{d_{B}}^{3}\beta^{2}\left( 1-\beta\right)}.$ | (S14) |
| --- | --- | --- |

The maximum tensile strain can be found using Eqn. (S6)

|  | $\varepsilon_{max}=\frac{2d_{B}v_{max}}{9\beta\left( 1-\beta\right)L^{2}}.$ | (S15) |
| --- | --- | --- |

**Table 3.** Mechanical property calculations for the uniform cylinder and tapered cylindrical beam models. For the *D. holocanthus* spines, *β* ~ 0.21. Maximum tensile stresses were obtained from Eqns. (S5) and (S14), Young’s moduli were obtained from Eqns. (S3) and (S10), maximum tensile strains were obtained from Eqns. (S6) and (S15).

|  | Uniform cylindrical beam | Tapered beam |
| --- | --- | --- |
| Maximum tensile stress | $\sigma_{max}=\frac{32 PL}{\pi d^{3}}$ | $\sigma_{max}=\frac{128PL}{27\pi{d_{B}}^{3}\beta^{2}\left( 1-\beta\right)}$ |
| Young’s modulus | $E=\frac{64PL^{3}}{3{\pi v}_{max}d^{4}}$ | $E =\frac{64 PL^{3}}{3\pi\beta{d_{B}}^{4}v_{max}}$ |
| Maximum tensile strain | $\varepsilon_{max}=\frac{3v_{max}d}{2L^{2}}$ | $\varepsilon_{max}=\frac{2v_{max}d_{B}}{9\beta\left( 1-\beta\right)L^{2}}$ |

*σ_max_* = maximum tensile stress, *P* = load on the beams, *L* = length of the beam, *d* = diameter of the uniform beam *v_max_* = maximum deflection of the beam, *d_B_* = large diameter of the tapered beam, and *β* = ratio between the smaller diameter and larger diameter of the tapered beam.

1. Derivation of Uniform Beam Diameter

The volume of the tapered beam is:

|  | $V_{tapered}=\frac{\pi L}{12}\left( {d_{A}}^{2}+d_{A}d_{B}+{d_{B}}^{2} \right).$ | (S16) |
| --- | --- | --- |

The volume of the uniform cross-section beam is:

|  | $V_{uniform}=\pi\left( \frac{d}{2} \right)^{2}L.$ | (S17) |
| --- | --- | --- |

Setting Eqn. (S16) equal to Eqn. (S17), one obtains

|  | $d=\sqrt{\frac{{d_{A}}^{2}+d_{A}d_{B}+{d_{B}}^{2}}{3}}.$ | (S18) |
| --- | --- | --- |
